# Contamination Evaluation and Source Identification of Heavy Metals in the Sediments from the Lishui River Watershed, Southern China

**DOI:** 10.3390/ijerph16030336

**Published:** 2019-01-25

**Authors:** Fang Shen, Longjiang Mao, Runxia Sun, Jijing Du, Zhihai Tan, Min Ding

**Affiliations:** 1 School of Marine Sciences, Nanjing University of Information Science and Technology, Nanjing 210044, China; mlj1214@nuist.edu.cn (F.S.); Sunrunxia@nuist.edu.cn (R.S.); djijing@163.com (J.D.); 2 Jiangsu Research Center for Ocean Survey Technology, Nanjing University of Information Science and Technology, Nanjing 210044, China; 3 Environmental and Chemical Engineering College, Xi’an Poly-technic University, Xi’an 710048, China; tonishtan@163.com; 4 School of Tourism, Taishan University, Tai’an 271021, China; jndm2612@sohu.com

**Keywords:** heavy metals, surface sediments, contamination evaluation, source identification, the Lishui River

## Abstract

Seven heavy metals (Cr, Mn, Co, Ni, Cu, Zn, Pb) were measured in surface sediments from the Lishui River watershed, an area with increased soil erosion in China. The mean concentrations of heavy metals were 61.20 mg/kg (Cr), 757.15 mg/kg (Mn), 9.39 mg/kg (Co), 25.31 mg/kg (Ni), 22.84 mg/kg (Cu), 91.66 mg/kg (Zn), and 40.19 mg/kg (Pb), respectively. The spatial distribution of heavy metals was site-specific, exhibiting a remarkably high level in the sampling stations with intense agricultural activities (Lixian) and industrial activities (Jinshi). Contamination indexes including contamination factor, pollution load index, nemerow multi-factor index, potential ecological risk index, and human health risk were used to assess the pollution degree of the river sediments. The results indicated the pollution degree of heavy metals decreased in the order of Mn > Pb > Zn > Cr > Cu > Ni > Co. Heavy metals resulted in non-pollution to moderate pollution, with low ecological risk and an acceptable carcinogenic risk caused by Cr and Ni for children and adults. Person’s correlation analysis and principal component analysis, coupled with cluster analysis, revealed that the sediments from the Lishui River were mainly influenced by two sources. Cr, Co, Ni, and Cu were mainly derived from natural sources, while Mn, Zn, and Pb originated from agricultural and industrial activities, mining, and vehicular traffic.

## 1. Introduction

According to statistics, more than 99% of heavy metals entering aquatic system can be stored in the sediment indifferent ways [1]. River sediments are important repositories, sinks, and carriers for heavy metals [2,3]. They play an important role in assessing metal contamination and tracing contamination sources [4]. Heavy metals can inflict serious environmental damage in the surroundings due to their toxicity, persistence, and bioaccumulation [3]. Following the frequency of natural and anthropogenic activities and the discharge of heavy metals into rivers, contamination is ubiquitous in the aquatic environment [5]. The natural sources of heavy metals mainly include geological weathering, soil erosion, and airborne dust [6]. Anthropogenic activities are the major cause of heavy metal pollution in the aquatic environment, such as industrial discharges, vehicular exhaust, mining operations, agricultural cultivation, and atmospheric precipitation [6,7]. Heavy metals drill through multiple biogeochemical cycles and eventually enter the human food chain, which brings about bioaccumulation and bio-magnification, and poses a potential threat to human health [8,9,10]. Heavy metal pollution, a widespread global problem, has attracted great concern due to the increasing concern of human health [9]. Therefore, it is essential to study the concentration, distribution, influence, and sources of heavy metals in the sediment to protect human health and environment.

The Lishui River is one of the four major rivers (Lishui River, Xiangjiang River, Zishui River, and Yuanjiang River) in Hunan Province, Southern China. The Lishui River basin is a densely populated area with a population of nearly 3.91 million and a significant agricultural production base in the national, especially in the Liyang plain [11,12]. The Liyang plain, situated on the northwestern bank of Dongting Lake, has crisscrossing rivers mainly composed of the Lishui River and its branches (the Danshui River and Censhui River) [13]. The Lishui River is vital for agricultural and domestic water supply in this area [12]. However, the environmental pressure of pollutants increasing with the economy of the Lishui River basin has achieved a steady and rapid growth in recent years [14,15]. The Lishui River flows through several agricultural and industrial areas and finally enters Dongting Lake. Severe heavy metal pollution has been found in the sediment from Dongting Lake [16]. The Lishui River is both the most sand-filled and the most serious soil erosion river among the Dongting Lake water system [17]. Hence, it is indispensable to study the heavy metal pollution in the sediments from the Lishui River.

The principal aims of this research are to (1) measure the contamination and investigate the distribution of seven heavy metals (Cr, Mn, Co, Ni, Cu, Zn, and Pb), (2) use sediment quality indicators including the contamination factor, the pollution load index, and the nemerow multi-factor index to assess the pollution degree of river sediments, (3) use potential ecological risk and health index to assess the risk of heavy metals, and (4) use multivariate analysis to distinguish the possible sources of heavy metals.

## 2. Materials and Methods

### 2.1. Background of Study Area

**Figure 1:**
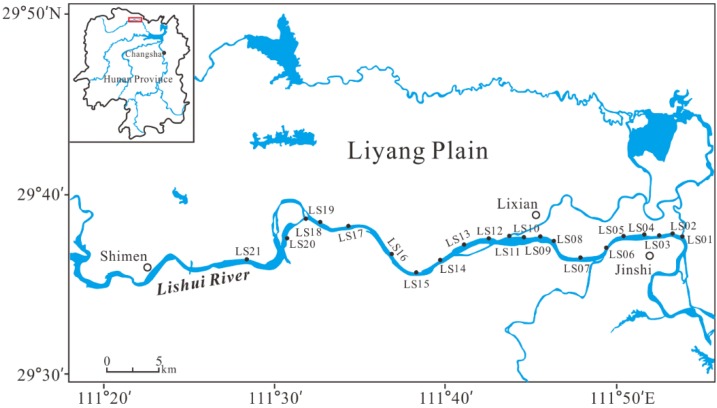
Sediment sampling stations in the Lishui River, China.

The Lishui River (29°30′–30°12′ N, 109°30′–112°00′ E), located in the northwest of Hunan Province, is the fourth largest river in the Dongting Lake basin [14]. The Lishui River originates from the Chinese fir community in Sangzhi County and flows through six counties (Sangzhi County, Dayong County, Cili County, Shimen County, Lixian County, and Jinshi County) from northwest and to southeast, and finally enters Dongting Lake [18,19]. The trunk steam and eight tributaries are collectively referred to as Jiuli. The Lishui River, with the spatial extent of 18,496 km^2^ and the main stream of 388 km, has an abundant and concentrated watercourse [20]. Its mean annual runoff is 1.70 × 10^10^ m^3^, and the natural drop is up to 1439 m, leading abundant hydropower resources [11]. The upper reach of the Lishui River flows through mountains and valleys, exposing storm runoff and bedrock; the middle reach flows through hills and basins; the downstream is plain and opening flat terrain, where population and economic activities are concentrated [12,19]. In the consideration of geology, population, and production, this research chose the downstream of the Lishui River as the study area. The study area covered Shimen County, Lixian County, and Jinshi County, situated in the south of the Liyang plain (Figure 1). This region features a subtropical climate, characterized by warm and humid summers, and cool and dry winters [11,13]. With an annual mean temperature of 16–17 °C, the annual mean precipitation is less than 1300 mm [11,13].

### 2.2. Sampling and Sample Preparation

Surface sediments were sampled from 21 locations across the Lishui River in the summer of 2016, as illustrated in Figure 1. Using a pre-cleaned and acid washed PVC spade, all sediment samples (0–5 cm) were immediately placed in acid-washed polyethylene bags. For the geochemical analysis of the sediments, all samples were transported to the laboratory and air-dried for two weeks to ambient temperature.

### 2.3. Analytical Methods

All of the samples for the geochemical analysis were powdered in an agate mortar [21]. The acid digestion method was applied for the extraction of heavy metals using HCl-HNO_3_-HF-HClO_4_. Fractions of approximately 100 mg of powdered sediments were digested to a mixture of 10 mL of HCl (ρ = 1.19 g/mL), 10 mL of HNO_3_ (ρ = 1.42 g/mL), 10 mL of HF (ρ = 1.49 g/mL), and 10 mL of HClO_4_ (ρ = 1.68 g/mL) digested at 180 °C in a microwave oven (ETHOS TOUCH CONTROL, Milestone Inc., Via Fatebenefratelli, 1/5 24010 Sorisole (BG), Italy) [22]. The obtained suspension liquid was then filtered using a membrane filter [8]. The concentrations of Cr, Mn, Co, Ni, Cu, Zn, and Pb in the sediment from the Lishui River were quantified with high-resolution inductively coupled plasma mass spectroscopy (HR-ICP-MS, Element II) at the State Key Laboratory for Deposits Research of Nanjing University. In order to guarantee the accuracy of the results, adequate quality assurance/quality control (QA/QC) was adopted in all aspects of the study [23]. The accuracy of the analysis was controlled by using reagent blanks, duplicate samples, and certified geochemical reference materials with a deviation of <5%.

### 2.4. Heavy Metal Concentration and Contamination Assessment

#### 2.4.1. Contamination Factor

The contamination factor (*CF*), a single index, is considered to be a simple and effective tool in monitoring the heavy metal contamination [24]. *CF* is calculated using the following equation:

$$CF_{i}=C_{i}/B_{i}$$

where *C_i_* and *B_i_* are the measured concentration and the background value of metal *i*, respectively. *CF_i_* is a contamination factor of heavy metal in the sediments. The contamination factor distinguishes four classes of quality for sediments as given in Table 1 [24]. The background concentrations of Cr, Mn, Co, Ni, Cu, Zn, and Pb are 71.3, 459.0, 14.6, 31.9, 27.3, 94.4, and 29.7 mg/kg [25].

#### 2.4.2. Pollution Load Index

The pollution load index (*PLI*) has been used to assess the comprehensive level of heavy metals for site, zone, or estuary [26]. The *PLI* value for a site in the sediment is calculated with the formula

$$PLI=\sqrt[{n}]{\prod_{i=1}^{n}CF_{i}},CF_{i}=C_{i}/B_{i}$$

where *CF_i_* is the ratio between the measured concentration (*C_i_*) and the background value (*B_i_*) of the heavy metal *i*; *n* is the number of heavy metals. *PLI* > 1 means polluted and *PLI* < 1 implies unpolluted [26].

#### 2.4.3. Nemerow Multi-Factor Index

The nemerow multi-factor index (*PI*), taking into consideration the most polluting factors in particular, can be used to assess the status of comprehensive pollution caused by all the heavy metals in the sediments, because different heavy metals may have impacts in the same station [27]. The nemerow multi-factor index (*PI*) is defined as

$$PI=\sqrt[{2}]{\frac{{\left(CF_{i\max}\right)}^{2}+{\left(CF_{iave}\right)}^{2}}{2}},CF_{i}=C_{i}/B_{i}$$

where *CF_i_* is the ratio between the measured concentration (*C_i_*) and background value (*B_i_*) of the heavy metal *i*; *CF_imax_* and *CF_iave_* represent the maximum contamination and average of contamination factors, respectively. *PI* is the nemerow multi-factor index, whose values are categorized as follows: *PI* < 1: unpolluted; 1 ≤ *PI* < 2.5: lowly polluted; 2.5 ≤ *PI* < 7: moderately polluted; *PI* ≥ 7: highly polluted [28].

#### 2.4.4. Potential Ecological Risk Index

The potential ecological risk index (*RI*) is used to assess the potential ecological risk of a given contaminant in sediments according to the toxicity of heavy metals and response of the environment [24]. The formulas of potential ecological risk index described by Hankanson are expressed as

$$C_{r}^{i}=C_{f}^{i}/C_{n}^{i},E_{r}^{i}=T_{r}^{i}\times C_{r}^{i},RI=\sum E_{r}^{i}$$

where $C_{r}^{i}$ is the pollution factor of the heavy metal *i*; $C_{f}^{i}$ and $C_{n}^{i}$ are the measured concentration and background value, respectively; $T_{r}^{i}$ is the toxic-response factor for a given substance (i.e., Cr = 2, Mn = Zn = 1, Co = Ni = Cu = Pb = 5); $E_{r}^{i}$ is the potential ecological risk index of the metal *i*; *RI* represents the potential ecological risk index of all heavy metals for a region, which is the sum of individual potential factor. The $E_{r}^{i}$ and *RI* are divided into five and four classes depending on the apart values given in Table 1, respectively [24].

### 2.5. Potential Human Health Risk

Human risk assessment is generally used to estimate the risk caused by the heavy metals of a known amount for human beings after chemical exposure [29]. Health risk assessment can be determined by the non-carcinogenic and carcinogenic risk for both adults and children. Hazard identification, exposure assessment, dose–response assessment, and risk characterization are the key elements of health risk assessment [30]. To predict the human health risk caused by the exposure of heavy metals, chronic daily intake (*CDI*) (mg/kg/day) through incidental ingestion (*CDI_ingest_*) and dermal contact (*CDI_dermal_*) was determined by the following formulas [31]:

$$\begin{array}{l} CDI_{ingest}=\frac{CS\times IR\times CF\times FI\times EF\times ED}{BW\times AT}\\ CDI_{dermal}=\frac{CS\times CF\times SA\times AF\times ABS\times EF\times ED}{BW\times AT} \end{array}$$

where *CS* is the heavy metal concentration in the sediment (mg/kg); *IR* is the ingestion rate (mg/day); *CF* is the conversion factor (kg/mg); *FI* is the fraction ingested from the contaminated source (unitless); the *EF* of the *CDI_ingesti_* is the exposure frequency (days/year); *ED* is the exposure duration (years); *BW* is the average body weight (kg); *AT* is the average time (days); *SA* is the exposed surface area of skin (cm^2^/event); *AF* is the skin adherence factor (mg/cm^2^); *ABS* is the dermal absorption factor (unitless); *EF* of *CDI_dermal_* is the exposure frequency (events/year). The values of the input parameters used to calculate *CDI* are given in Table 2.

#### 2.5.1. Non-Carcinogenic Risk

Hazard index (*HI*) is characterized by the sum of hazard quotients (*HQ*), indicating the cumulative non-carcinogenic. *HQ* represents the ratio of the chronic daily intake (*CDI*) and the corresponding reference dose (*RfD*). The *RfD* values of Cr, Mn, Co, Ni, Cu, Zn, and Pb are 0.003, 0.14, 0.02, 0.02, 0.0371, 0.3, and 0.0035 mg/kg/day [33]. A value of *HI* below 1 means no significant non-carcinogenic risk; a value of *HI* surpassing 1 indicates adverse non-carcinogenic risk effects may occur [32]. *HQ* and *HI* are expressed as follows [31]:

$$\begin{array}{l} HQ=CDI/RfD\\ HI=\sum HQ=HQ_{ingest}+HQ_{dermal} \end{array}$$

#### 2.5.2. Carcinogenic Risk

Carcinogenic risk (*CR*) is estimated with the product of the chronic daily intake (*CDI*) and the cancer slope factor (*CSF*) over a lifetime. The cancer slope factor (*CSF*) plays a key role in convention that the daily toxin intake changes into the incremental risk of an individual developing cancer. Due to the lake of *CSF* values, only the carcinogenic risk of Cr, Ni, and Pb was estimated. The *CSF* values of Cr, Ni, and Pb are 0.5, 0.84, and 0.0085, respectively [33]. *CR* through an individual exposure pathway can be summed to generate the total cancer risk (*TCR*). A value of *TCR* ranging from 1 × 10^−6^ to 1 × 10^−4^ is considered an acceptable or tolerant scope [33]. A *TCR* less than 1 × 10^−6^ doesnot have dramatic effects on human health, while a *TCR* exceeding 1 × 10^−4^ is regarded as unacceptable [33]. *CR* and *TCR* are calculated by the following equations [31]:

$$\begin{array}{l} CR=CDI\times CSF\\ TCR=\sum CR=CR_{ingest}+CR_{dermal} \end{array}$$

### 2.6. Multivariate Analysis

Multivariate analysis, including Pearson’s correlation analysis, principal component analysis (PCA), and cluster analysis (CA), is an effective tool to identify the sources of heavy metals [34,35]. Pearson’s correlation analysis is used to evaluate the relativity between heavy metals elements. Principal component analysis (PCA) is used to reduce the dimension of data and extract highly correlated heavy metal elements into independent factors. Cluster analysis (CA) classifies heavy metal elements into different classes, and the classes required for clustering are unknown. Multivariate analysis was carried out using the SPSS statistical package.

## 3. Results and Discussion

### 3.1. Heavy Metal Concentration and Spatial Distribution

**Figure 2:**
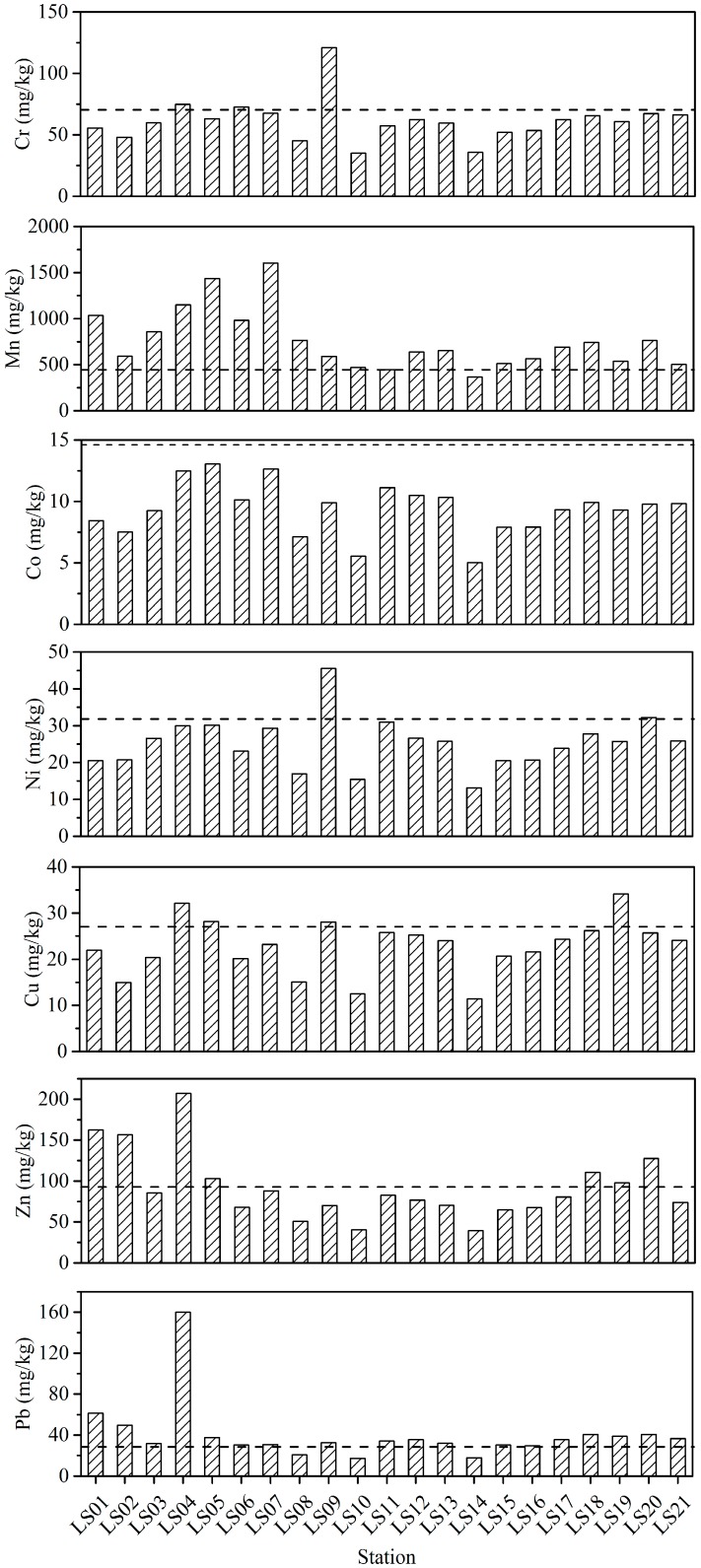
Heavy metals concentrations (mg/kg) in the sediment from the Lishui River.

Figure 2 presented the concentrations of heavy metals (Cr, Mn, Co, Ni, Cu, Zn, and Pb) in the sediment collected from the Lishui River. The concentrations of Cr, Mn, Co, Ni, Cu, Zn, and Pb ranged from 35.01 to 120.94, from 366.66 to 1604.32, from 5.04 to 13.05, from 13.14 to 45.54, from 11.40 to 34.13, from 39.56 to 207.16, and from 17.25 to 159.91 mg/kg, respectively. The mean concentrations of the studied heavy metals descended in the order of Mn (757.15 mg/kg)>Zn (91.66 mg/kg)>Cr (61.20 mg/kg)>Pb (40.19 mg/kg)>Ni (25.31 mg/kg)>Cu (22.84 mg/kg)>Co (9.39 mg/kg). The concentrations of the Lishui River were compared with the concentrations reported from other rivers in China and other countries (Table 3). The trend was the same as that in the Zishui River flowing into the Dongting Lake, but different from that in the Xiangjiang River [36]. The levels of heavy metal in the present study were lower than those in the Zishui River and the Xiangjiang River [36,37]. However, the concentrations of heavy metals in the Lishui River were higher than those in the rivers in Guangdong, one of the most developed regions in China [5]. The concentrations of heavy metals (Cr, Mn, Co, Ni, Cu, and Zn) in the Lishui River were higher than the values investigated in the Zahuapan River in Mexico, a reference river in eco-toxicological studies [38]. The characteristics of heavy metal concentrations had a big difference between the Lishui River and the rivers in Mexico (Zahuapan River, Atoyac River). The concentration of Mn in the Lishui River was much larger than that of the Zahuapan River and the Atoyac River, while the concentrations of Cr and Pb in the Lishui River were much lower than the concentrations in the Zahuapan River and the Atoyac River [39].

The highest concentrations of Cr and Ni were found at Station LS09 (120.94 mg/kg for Cr and 45.54 mg/kg for Ni, respectively). The levels of Cr and Ni in the other sampling sites exhibited slight fluctuations, which were comparable to lower than the respective background values [25]. Sampling Station LS09 is located in Lixian, an important agricultural production base in China. Intensive agricultural activity may be a plausible reason. Previous studies have demonstrated that the abuse of pesticides and phosphate fertilizer in agriculture caused cadmium deposition in the sediments [40]. Elevated levels of Mn, Zn, and Pb were all observed in the sampling stations next to Jinshi, a developed industrial city in China. Co and Cu both showed similar concentrations among sampling sites.

### 3.2. Heavy Metal Contamination Assessment

**Figure 3:**
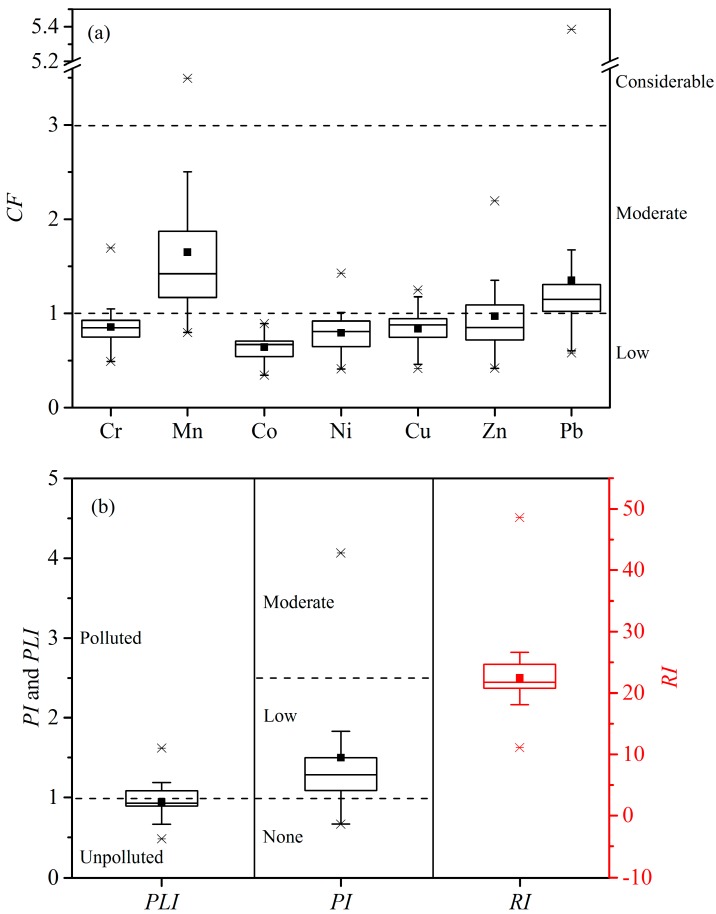
*CF*, *PLI*, *PI,* and *RI* values of heavy metals in the sediment from the Lishui River. (**a**) *CF* values; (**b**) *PLI*, *PI* and *RI* values.

In order to comprehensively understand the contamination of heavy metals in the sediments of the Lishui River, the contamination factor, pollution load index, and nemerow multi-factor index were applied to evaluate the pollution levels in the present study (Figure 3). The average *CF* values were 0.86 for Cr, 1.65 for Mn, 0.64 for Co, 0.79 for Ni, 0.84 for Cu, 0.97 for Zn, and 1.35 for Pb, respectively. The results indicated that, overall, the surface sediments in the Lishui River were moderately polluted by Mn and Pb. In contrast, Cr, Ni, Cu, Zn, and Co exhibited low concentration. However, it is noteworthy that several sampling sites showed considerable contamination of Mn (LS05 and LS07) and Pb (LS04), and moderate contamination of Cr (LS09), Ni (LS09), Cu (LS09 and LS19), and Zn (LS01, LS02, LS04, LS05, LS18, and LS20). The *PLI* and *PI* values reflected the status of comprehensive pollution caused by all the heavy metals. The *PLI* and *PI* ranged from 0.48 to 1.62 and from 0.67 to 4.06, respectively. This implied heavy metal pollution existed in the sediments from the Lishui River, which was consistent with the results obtained from *CF* values. The values of *PLI* indicated that the stations with values greater than 1, expressing the sediment polluted by heavy metals, were LS01, LS04, LS05, LS07, LS09, and LS20. And the three worst stations of heavy metal pollution were LS04 (1.62), LS05 (1.19), and LS07 (1.12). The values of *PI* exhibited that Stations LS04 (4.07) and LS07 (2.64) were both between 2.5 and 7, meaning moderately polluted, while LS10, LS14, and LS15 were less than 1, indicating no pollution. The other stations were polluted by lowly by heavy metals on the basis of *PI* values. Hence, the values of *PLI* and *PI* both indicated that the sampling sites between the Jinshi section and the Lixian section of the Lishui River (LS04, LS05, and LS07) were the three heavy metal pollution stations. The pollution of Station LS04 mainly caused by the considerable contamination of Pb. Heavy metal contamination in Stations LS05 and LS07 resulted from the considerable contamination of Mn.

The results of potential ecological risk index were displayed in Figure 3b. The potential ecological factors $E_{r}^{i}$ of seven heavy metals (Cr, Mn, Co, Ni, Cu, Zn, and Pb) were all less than 40. Moreover, the *RI* values for all stations were all less than 95. All studied heavy metals posed a low potential ecological risk at the sediment from the Lishui River, and all sampling sites exhibited a low potential ecological risk of heavy metals.

**Figure 4:**
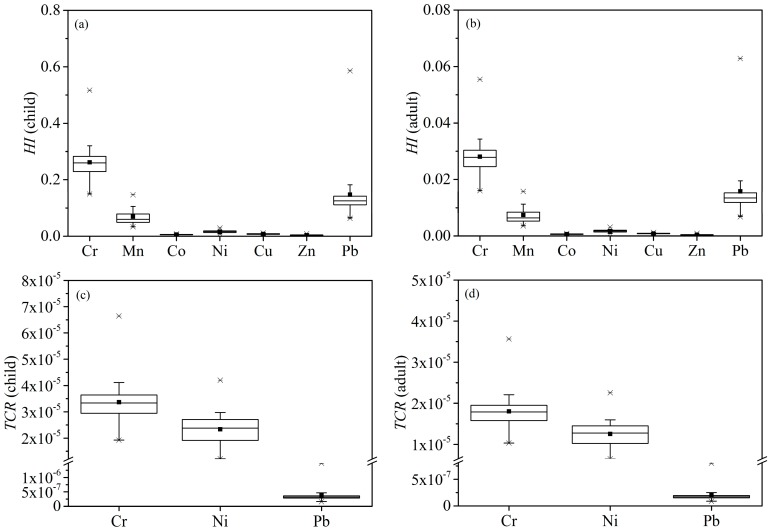
*HI* and *TCR* for child and adult of heavy metals. (**a**) *HI* for child; (**b**) *HI* for adult; (**c**) *TCR* for child; (**d**) *TCR* for adult.

Human health risk caused by heavy metals was assessed by the hazard index (*HI*) for non-carcinogenic risk and the total cancer risk (*TCR*) value for carcinogenic risk for both children and adults (Figure 4). The *HI* values of the studied heavy metals ranged from 0.0017 to 0.5858 for children and from 0.0002 to 0.0628 for adults, respectively. *HI* values for children and adults were investigated in the descending order of Cr>Pb>Mn>Ni>Cu>Co >Zn. The results suggested that there would not be a non-carcinogenic risk caused by heavy metals for children and adults, although the *HI* values for children were an order of magnitude higher than that for adults.

The *TCR* values of Cr, Ni, and Pb on children were 1.92 × 10^−5^–6.65 × 10^−5^ (3.36 × 10^−5^, mean), 1.21 × 10^−5^–4.20 × 10^−5^ (2.34 × 10^−5^) and 1.61×10^−7^–1.49 × 10^−6^ (3.75 × 10^−7^), respectively. For adults, the *TCR* values of Cr, Ni, and Pb were 1.80 × 10^−5^, 1.25 × 10^−5^, and 2.01 × 10^−7^ in sequence. As the same with *HI* values, the *TCR* values of adults were smaller than the children’s values. The maximum acceptable risk level of 1 × 10^−4^ is set by the US EPA, while a *TCR* less than 1 × 10^−6^ doesnot have dramatic effects on human health. The average *TCR* values for children and adults suggested that these carcinogenic risk levels caused by Cr and Ni were acceptable, and Pb might pose no carcinogenic risk. These findings demonstrated that both children and adults were more easily exposed to Cr and Ni than Pb, which may cause an acceptable carcinogenic risk.

### 3.3. Identification of Pollution Sources

**Figure 5:**
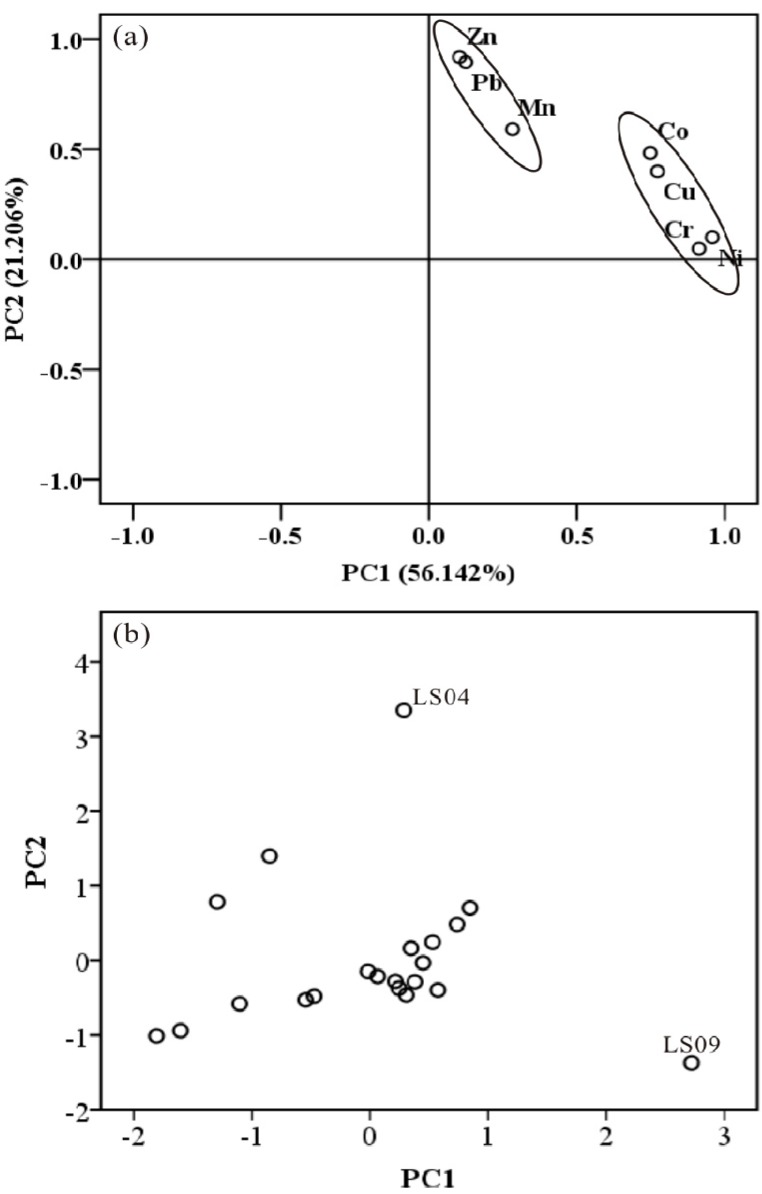
The principal component analysis results heavy metals. (**a**) Factor loadings; (**b**) factor scores.

**Figure 6:**
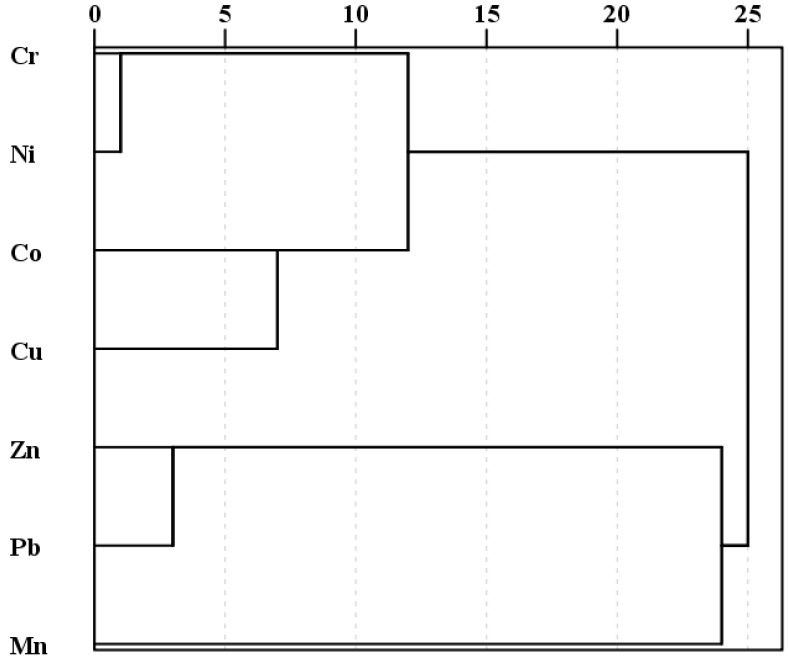
Dendrogram of cluster analysis for heavy metals in the sediment from the Lishui River.

Multivariate statistical analysis, including Pearson’s analysis, principal component analysis, (PCA) and cluster analysis (CA), could be used to identify similar origins or geochemical characteristics between the heavy metals when they have inter-relationship [34,35]. The closeness of the relationship between heavy metals Cr, Mn, Co, Ni, Cu, Zn, and Pb could be revealed by Pearson’s correlation analysis (Table 4). The Pearson’s correlation coefficients of elemental pairs Cr–Co (0.569), Cr–Ni (0.906), Cr–Cu (0.627), Co–Ni (0.707), Co–Cu (0.769), and Ni–Cu (0.725) implied that a significant correlation at *p* < 0.01 was found among Cr, Co, Ni, and Cu. Mn exhibited a significant correlation with Co, and an in apparent correlation with other metals (Ni, Cu, Zn, and Pb). A significant positive correlation at *p* < 0.01 was also observed between Zn and Pb. Principal component analysis showed that two principal components with an eigenvalue>1 occupied appropriately 77.348% of the total variance (Figure 5a). The first principal component was dominated by Cr, Co, Ni, and Cu, explaining 56.142% of the total variance. The second component was loaded by Mn, Zn, and Pb, accounting for 21.206% of the total variance. The factor score plot showed that Station LS04 had a high score in PC2, indicating considerable contamination of Pb, and moderate contamination of Mn and Zn (Figure 5b). Station LS09, moderately polluted by Cr, Ni, and Pb, had a high score in PC1. The between-group linkage was applied to cluster analysis, and Pearson’s correlation for similarity in heavy metals was calculated. A dendrogram of cluster analysis helped to group the heavy metals with characteristics and to allow the two groups to be distinguished from one another (Figure 6). Cluster1 contained Cr, Ni, Co, and Cu; Cluster2 consisted of the remaining three metals: Mn, Zn, and Pb.

The results of multivariate statistical analysis reached a consensus, indicating that the studied heavy metals were divided into two clusters: (1) Cr, Ni, Co, and Cu; (2) Mn, Zn, and Pb. The concentrations of the first species (Cr, Ni, Co, and Cu) were close to the corresponding background values, which implies that these heavy metals were mainly derived from natural sources. The concentrations of the second species of heavy metals were greater than the corresponding values, which revealed human activities was a major influence. The Zn concentration of the six stations (LS01, LS02, LS04, LS05, LS18, and LS20) exceeded the background concentration. Zn infiltrated the aquatic system due to petrochemical and industrial effluents, vehicular traffic, fertilizer, and livestock manure [7,41]. Stations LS01, LS02, LS04, and LS05 were located in the Jinshi County. The livestock and poultry industry produced a considerable quantity of manure in Jinshi County, which was a major source of agricultural pollution [42]. All stations polluted by Zn were located in the Liyang plain. Being the origin of world rice farming, Liyang plain’s agriculture was advanced, and the environment was polluted by fertilizer and other farm runoff [43]. The moderate pollution of Mn was investigated in the sediments of the Lishui River. The sources of Mn were attributed to agricultural and industrial activities, including coal combustion and coal mining [7]. Pollutants produced by agricultural activities in the Liyang plain, such as the employment of pesticides, were discharged into the Lishui River [44]. Many different mine dimensions were distributed in the Lishui River basin, containing nonferrous and ferrous metals [45,46]. According to statistics, there were 206 mine enterprises being mined, including open pit mining enterprises and underground mining enterprises [45]. The mining of these minerals brought elements such as Mn, Pb, and Hg into the sediment [46]. Pb primarily came from metal plating, vehicle exhaust, fertilizer, and wastewater discharge [41,47]. Industrial estates of Jinshi County, Lixian County, and Shimen County all lay near the Lishui River. Jinshi has been a famous industry center in Hunan Province since the 1970s, and this district’s industrial concentration achieved a provincial level. The economy is dominated by this industry. Enterprises, such as the generation of electricity and the manufacturing industry, discharged wastewater during production. The Liyang plain’s agricultural activities releases fertilizers into the Lishui River. The area from Shimen to Xiaodukou in the Lishui River was the best navigable reach, and ships under 100t can navigate there throughout the year, and this has caused the pollution of Pb.

## 4. Conclusions

Heavy metals (Cr, Mn, Co, Ni, Cu, Zn, and Pb) were measured in the surface sediments from the Lishui River. The mean concentrations of heavy metals were 61.20 mg/kg (Cr), 757.15 mg/kg (Mn), 9.39 mg/kg (Co), 25.31 mg/kg (Ni), 22.84 mg/kg (Cu), 91.66 mg/kg (Zn), and 40.19 mg/kg (Pb), respectively. The spatial distribution of heavy metals was site-specific, exhibiting a remarkably high level in the sampling stations with intense agricultural activities (Lixian) and industrial activities (Jinshi). To investigate the contamination degree, ecological risk, and human health risk of heavy metals in the sediment, contamination factor (*CF*), pollution load index (*PLI*), nemerow multi-factor index (*PI*), potential ecological risk index (*RI*), and human health risk were employed to assess the pollution degree, potential ecological risk, and human health risk of the river sediments. The results showed that the degree of heavy metals decreased in the order of Mn>Pb>Zn>Cr>Cu>Ni>Co. Heavy metals resulted in non-pollution to moderate pollution, with low ecological risk and an acceptable carcinogenic risk caused by Cr and Ni for children and adults. Person’s correlation analysis, principal component analysis (PCA), and cluster analysis (CA) were applied to identify the sources of heavy metal pollution. The results indicated the sediment from the Lishui River were mainly influenced by two sources: Cr, Mn, Ni, and Cu were mainly derived from natural sources, while Mn, Zn, and Pb were originated from agricultural and industrial activities, mining, and vehicular traffic.

## Figures and Tables

**Table 1:** The classes of contamination factor (*CF*) and potential ecological risk [24,26].

CF	Contamination Degree	$E_{r}^{i}$	RI	Potential Ecological Risk
CF_i_ < 1	Low	$E_{r}^{i}$ < 40	RI < 150	Low
1 ≤ CF_i_ < 3	Moderate	40 ≤ $E_{r}^{i}$ < 80	150 ≤ RI < 300	Moderate
3 ≤ CF_i_ < 6	Considerable	80 ≤ $E_{r}^{i}$ < 160	300 ≤ RI < 600	Considerable
CF_i_ ≥ 6	Very high	160 ≤ $E_{r}^{i}$ < 320		High
		$E_{r}^{i}$ ≥ 320	RI ≥ 600	Very high

**Table 2:** The values of parameters used to estimate chronic daily intake (*CDI*).

Parameter	Value
*IR*	Child: 200 mg/day; adult: 100 mg/day
*CF*	1 × 10^−6^ kg/mg
*FI*	1.0
*EF*	350 days/year
*ED*	Child: 6 years; adult: 30 years
*BW*	Child: 15 kg; adult: 70 kg
*AT*	Non-carcinogenic: ED × 365 days/years; carcinogenic: 70 years × 365 days/years
*SA*	Child: 2800 cm^2^; adult: 5700 cm^2^
*AF*	Child: 0.2; adult: 0.07
*ABS*	0.001
*EF*	1 events/day × 350 days/year

Adapted from US Environmental Protection Agency [31,32].

**Table 3:** Heavy metal concentrations in the sediments from the Lishui River and other selected rivers.

Name of the River, Country	Heavy Metal Concentrations (mg/kg)							References
	Cr	Mn	Co	Ni	Cu	Zn	Pb	
Lishui River, China	61.20	757.15	9.39	25.31	22.84	91.66	40.19	This study
Zishui River, China	67.51	1322.89	16.76	34.66	34.19	141.90	35.68	[36]
Xiangjiang River, China	120.44	1805.17	23.19	57.14	101.36	443.32	214.91	[37]
Rivers in Guangdong Province, China	21.81	325.78	4.58	15.99	15.71	60.16	26.93	[5]
Zarrin-Gol River, Iran	37.67	286.28	8.79	12.39	-	32.68	-	[38]
Zahuapan River, Mexico	121.63	293.88	8.25	19.38	12.63	91.63	9.00	[39]
Atoyac River, Mexico	181.83	158.17	5.33	22.00	14.17	62.17	12.17	[39]
Background value	71.30	459.00	14.60	31.90	27.30	94.40	29.70	[25]

**Table 4:** Person’s correlation matrix of the heavy metals in the sediments from the Lishui River.

Heavy Metal	Cr	Mn	Co	Ni	Cu	Zn	Pb
Cr	1						
Mn	0.233	1					
Co	0.569 **	0.674 **	1				
Ni	0.906 **	0.259	0.707 **	1			
Cu	0.627 **	0.283	0.769 **	0.725 **	1		
Zn	0.193	0.417	0.414	0.258	0.442 *	1	
Pb	0.243	0.34	0.423	0.233	0.476 *	0.841 **	1

* Correlation is significant at *p* < 0.05 (two-tailed); ** Correlation is significant at *p* < 0.01 (two-tailed).
